# Oxysophocarpine suppresses hepatocellular carcinoma growth and sensitizes the therapeutic blockade of anti‐Lag‐3 via reducing FGL1 expression

**DOI:** 10.1002/cam4.3151

**Published:** 2020-08-18

**Authors:** Jianchu Wang, Wang Wei, Qianli Tang, Libai Lu, Zongjiang Luo, Wenchuan Li, Yuan Lu, Jian Pu

**Affiliations:** ^1^ Department of Hepatobiliary Surgery Affiliated Hospital of Youjiang Medical University for Nationalities Baise China; ^2^ Clinic Medicine Research Center of Hepatobiliary Diseases Affiliated Hospital of Youjiang Medical University for Nationalities Baise China

**Keywords:** anti‐Lag‐3, FGL1, hepatocellular carcinoma, oxysophocarpine

## Abstract

Hepatocellular carcinoma (HCC) is an aggressive malignancy with limited effective treatments and ranks as the second most lethal tumor. Immunotherapy has brought great hope for HCC treatment. Oxysophocarpine is a bioactive alkaloid which poses various pharmacological functions including neuroprotective, anti‐virus, anti‐convulsant, and anti‐nociception. However, there is little systematic study of Oxysophocarpine against HCC and its underlying potential and mechanism combined with immunotherapy in HCC treatment remain poorly unknown. This study was aimed to investigate whether Oxysophocarpine can distinctly suppress HCC cells and sensitize the immunotherapy of CD8^+^ T cells against HCC. We used HepG2, Hepa1‐6, and primary CD8^+^ T cells to perform in vitro assays and Hepa1‐6 subcutaneous tumor to conduct in vivo assay. Oxysophocarpine inhibited the proliferation and increased the apoptosis of HepG2 and Hepa1‐6 cells, meanwhile suppressed the migration of HepG2 and Hepa1‐6 cells. Oxysophocarpine sensitized the Lag‐3 immunotherapy effect of CD8^+^ T cells against HCC in vivo and in vitro by decreasing Fibrinogen‐like protein 1 (FGL1) expression through downregulating IL‐6‐mediated JAK2/STAT3 signaling, whereas Oxysophocarpine treatment had a little effect of CD8^+^ T cells cytotoxicity function against HCC with PD‐1, Tim‐3, or TIGIT blockade. Our studies provided preclinical basis for clinical application of Oxysophocarpine.

## Graphical abstract

Oxysophocarpine inhibited the proliferation, migration, and increased the apoptosis of HCC cells. Oxysophocarpine sensitized the Lag‐3 immunotherapy effect of CD8^+^ T against HCC by decreasing FGL1 expression through IL‐6‐mediated JAK2/STAT3 pathway.

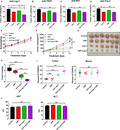

## 1 INTRODUCTION

Hepatocellular Carcinoma (HCC) is a common primary malignancy and comprises for 75%‐85% of primary liver cancer due to long‐term chronic liver disorder and inflammation.^1^ HCC ranks as the sixth most common cancer and the fourth leading cause of cancer death in the word.^2^ The incidence and mortality of HCC in men are 2 to 3 times higher than woman.^2^ The development of HCC is related to HBV or HCV inflection, alcohol abuse, aflatoxin‐contaminated dietary, smoking, obesity, and type 2 diabetes.^3^ For early stage HCC patients, liver transplantation, surgical resection, or radiation are suggested as the standard of care treatments.^4^ However, most HCC patients are diagnosed at advanced stages who are not suitable for liver transplantation or resection.^5^ Until now, there are only two systemic therapy (sorafenib and lenvatinib) approved by FDA for the first‐line advanced HCC treatments.^1^, ^6^ Hence, it is urgent to explore new therapeutic approaches for HCC treatment.

Recently, immunotherapy has becoming a promising treatment for cancer patients with remarkable clinical success such as anti‐PD1, anti‐PD‐L1, and anti‐CTLA‐4.^5^ Immune checkpoint inhibition blocks the negative regulatory signals either directly on T cells or on cells that interact with T cells.^7^ Among this, immune checkpoints of CD8^+^ T cells have been widely investigated as the target for immunotherapy such as PD‐1, Lag‐3, Tim‐3, TIGIT, and CTLA‐4.^8^ For advanced HCC, two anti‐PD1 monoclonal antibodies (pembrolizumab and nivolumab) have been approved as second‐line treatment therapy.^9^, ^10^ However, the clinical efficiency of immunotherapy varies widely in HCC due to resistance or therapy induced side effect such as poor liver function.^1^ Therefore, there is a need to develop more effective immune checkpoint inhibition therapies with limited underlying side effect.

Oxysophocarpine (OSP) is one of the main bioactive alkaloids extracted from *Sophora flavescens* Ait, *S alopecuroides L,* and other leguminous plants of the *Robinia*.^11^, ^12^ Oxysophocarpine is a traditional Chinese medicine, which poses various pharmacological functions including neuroprotective,^13^, ^14^ anti‐virus,^15^ anticonvulsant^12^ and anti‐nociception.^16^ In the anti‐tumor function, it has been identified that Oxysophocarpine has obvious suppressive effect against oral squamous cell carcinoma (Tca8113, Cal27, SCC‐9, SCC‐15, and SCC‐25 cell lines) and liver cancer (H22 cell lines).^11^, ^17^ However, the effect of Oxysophocarpine on HepG2 or Hepa1‐6 liver cancer cell lines and its underlying potential combined with immunotherapy in HCC treatment remain poorly unknown.

Here we identified that Oxysophocarpine suppressed the proliferation and migration of HepG2 and Hepa1‐6 cells, induced HepG2 and Hepa1‐6 cells apoptosis. In vivo and in vitro assays showed Oxysophocarpine sensitized the anti‐Lag‐3 immunotherapy effect against HCC by decreasing FGL1 expression through downregulating IL‐6‐mediated JAK2/STAT3 signaling, whereas Oxysophocarpine had a little effect of CD8^+^ T cells cytotoxicity function against HCC cells with TIGIT, PD‐1, or Tim‐3 blockade. These data provided evidence for the potential application of Oxysophocarpine against HCC.

## 2 MATERIALS AND METHODS

### 2.1 Material

HepG2 and Hepa1‐6 cell lines were bought from Cell Bank of Shanghai Institutes for Biological Sciences (China). All chemicals were of analytical grade. Sources for main chemicals and reagents were as follows: Oxysophocarpine was purchased from Dalian Meilun Biotechnology (cat#MB7000); Anti‐IL‐6R was obtained from selleck (cat#2012); 1640 medium (cat#C11875500CP, Gibco); DMDM medium (cat#C11995500CP, Gibco); Fetal bovine serum (cat#10099141, Gibco); penicillin‐streptomycin (cat#15140122, Gibco); 0.25% trypsin (cat#25200072, Gibco); Cell Counting Kit‐8 (cat#CK04, DOJINDO); FITC/Annexin V apoptosis detection Kit (cat#556547, BD Pharmingen).

### 2.2 Cell culture

HepG2 and Hepa1‐6 cell lines were cultured in DMEM medium supplemented with 10% FBS and 1% penicillin‐streptomycin and maintained in a humidified atmosphere containing 5% CO_2_ at 37℃. For each experiment, HepG2 and Hepa1‐6 cells were harvested using 0.25% trypsin.

### 2.3 Cell viability assay

Cell Counting Kit‐8 (CCK‐8) was used to examine the suppression effect of Oxysophocarpine on the viability of Hepa1‐6 and HepG2 cell lines according to the manufacturer's instruction. In brief, 1 × 10^4^/ well HepG2 or Hepa1‐6 cells were previously seeded into 96‐well plate for 24 hours before Oxysophocarpine treatment. Next, Hepa1‐6 and HepG2 cells were treated with different concentrations of Oxysophocarpine (0, 5, 10, and 20 µmol/L) and cultured for another 24, 48, or 72 hours. The concrete absorbance of each well was quantified by Microplate Reader at 450 nm wavelength.

### 2.4 Cell apoptosis assay

The FITC/Annexin V apoptosis detection Kit was applied to determine the apoptosis of HepG2 and Hepa1‐6 cells after Oxysophocarpine treatment according to the manufacturer's instructions. Briefly, HepG2 or Hepa1‐6 cells were previously seeded in six‐well plate at density of 1 × 10^6^ cells/well for 6 hours and then treated with different dosage Oxysophocarpine (0, 5, 10, and 20 µmol/L) for another 24 hours. HepG2 or Hepa1‐6 cells were harvested and re‐suspended in binding buffer at density of 1 × 10^6^ cells/ml. Next, transferring 100 μL cell solution into 1.5 mL centrifuge tube and adding 5 μL of FITC Annexin V and 5 μL PI into each tube. Thereafter, gently vortex the tube and incubating for 15 minutes at room temperature in the dark and adding 400 μL of binding buffer to each tube before analyzing by Beckman cytoflex S.

### 2.5 Cell migration assay

HepG2 and Hepa1‐6 cells migration assay were performed using 24‐trans well plates with 8‐μm diameter filters (cat#3422, Corning). HepG2 and Hepa1‐6 cells were previously treated with different dosage Oxysophocarpine (0, 5, 10, and 20 µmol/L) for 24 hours. Then 1 × 10^5^/ well Oxysophocarpine‐treated HepG2 or Hepa1‐6 cells were suspended in 200 μL serum‐free DMEM and placed in the upper chamber when the lower chambers were added 600 μL DMEM containing 10% FBS. The palate was maintained in a humidified atmosphere containing 5% CO_2_ at 37℃ for 24 hours. Then the filter was washed twice by PBS and fixed in 4% Paraformaldehyde Solution for 30 minutes, following stained with Crystal Violet Staining Solution for another 20 minutes. Last, the filter was washed with PBS to remove extra Crystal Violet Staining Solution. The migration cell images in the filter were collected under microscope at 200× magnification.

### 2.6 Tumor cell and CD8^+^ T cells coculture assay

EasySepTM Mouse CD8^+^ T‐cell Isolation Kit (cat#19853, Stemcell Technology) was used to isolate CD8^+^ T cells. First, mouse spleen was isolated from 8‐weeks‐old C57BL/6 mice and passed it through a 70‐μm cell strainer by syringe to produce single‐cell suspensions. After removing red blood cells, EasySepTM Mouse CD8^+^ T cell Isolation Kit was used to separate CD8^+^ T cell from single‐cell suspensions according to the manufacturer's instructions. CD8^+^ T cells were previously cultured in 1640 medium containing 10% FBS, 2.5 μg/mL anti‐CD3 (cat#16‐0031‐86, ebioscience) and 5 μg/mL anti‐CD28 (cat#16‐0281‐86, ebioscience) for 24 hours before cocultured with luciferase‐labeled Hepa1‐6 cells. 5000/well luciferase‐labeled Hepa1‐6 cells were planted into 96‐well culture plates and treated with 5 µmol/L Oxysophocarpine for 24 hours before cocultured with CD8^+^ T cells. Then, CD8^+^ T cells co‐cultured with Oxysophocarpine‐treated Hepa1‐6 cells with the ratio of 5:1 and 10 μg/mL Lag‐3, PD‐1, TIGIT, or Tim‐3 antibody (cat#BE0174, BE0146, BE0274, BE0115, BioXcell, USA) was added into coculture system for 48 hours at 37℃. To determine the lysis level of Hepa1‐6 cells, D‐luciferin was added into the 96‐well plates. Fluorescence microplate reader was used to detect the fluorescence values of each well.

### 2.7 Animal models

To establish subcutaneous tumor model, 1 × 10^6^ Hepa1‐6 tumor cells were planted into the left flanks of C57BL/6 mice (4 weeks, male). After 7 days, tumor‐bearing mice were dived into four groups including control, Oxysophocarpine, anti‐Lag‐3, and Oxysophocarpine combined with anti‐Lag‐3 groups. Tumor volume was measured by Vernier caliper and calculated by the formula 1/2 a × b^2^, where a was the long diameter and b was the short diameter. Mice were treated with 50mg/kg Oxysophocarpine (daily, intraperitoneally) or 100 μg anti‐Lag‐3 antibody per mouse (every 7 days, intraperitoneally). At 12 days after treatment, all mice were sacrificed.

### 2.8 Quantitative real‐time PCR

HepG2 or Hepa1‐6 cells were seeded into six‐well plates for 6 hours before treated with different concentration of Oxysophocarpine (0, 5, 10, and 20 µmol/L) for another 24 hours. Then, HepG2 or Hepa1‐6 cells were harvested and the total RNA was isolated from by Trizol reagent (cat#15596026, Invitrogen). Then RNA was reverse‐transcribed into cDNA with PrimeScript^TM^ RT reagent Kit (cat#RR037B, Takara) according to the manufacturer's protocols. RT‐PCR was performed on a 7900HT Fast Real‐Time PCR system (Applied biosystems) using SYBR green as the detection fluorophore. All values of target genes expression (Ct) were normalized to housekeeping gene GAPDH. The primer sequences were provided as follows: HepG2, FGL1 forward, 5′‑GCAAGGAGTCTGCTTCTGCT‑3′ and FGL1 reverse, 5′‑TGCCATGTTCCCCCTTGAAA‑3′; IL‐6 receptor forward, 5′‐ CCCCTCAGCAATGTTGTTTGT‐3′ and IL‐6 receptor reverse, 5′‐ CTCCGGGACTGCTAACTGG‐3′; GAPDH forward 5′‐AAGAAGG TGGTGAAGCAGGC‐3′ and GAPDH reverse, 5′‐TCCACCACCCT GTTGCTGTA‐3′. Hepa1‐6, FGL1 forward, 5′‑CCCTGTCAGGAACTTTTCATCC‑3′ and FGL1 reverse, 5′‑CGGTAGTAAACACCGTTCAGGT‑3′; GAPDH forward 5′‐ ATGTTCCAGTATGACTCCACTCAC‐3′ and GAPDH reverse, 5′‐ GACACAGTAGACTCCACGACATA‐3′.

### 2.9 Western blotting

HepG2 or Hepa1‐6 cells were treated with different concentration of Oxysophocarpine (0, 5, 10 and 20 µmol/L) for 24 hours. Then HepG2 and Hepa1‐6 cells were washed by cold PBS and lysed in RIPA buffer (cat#P0013, Beyotime Biotechnology) supplemented with protease inhibitor for 15 minutes on ice. The supernatants were collected after the lysates were centrifuged for 15 minutes on 12 000 *g* at 4℃. The total protein concentration of each sample was determined using BCA protein assay kit (cat#23225, Thermo Fisher Scientific). Protein extracts were separated by SDS‐PAGE gels, then transferred to PVDF membranes (cat#IPVH00010, Millipore). Membranes were blocked in 5% skimmed milk and then incubated with primary antibodies overnight at 4℃. The primary antibodies include FGL1 (1:1000, Proteintech, 16000‐1‐AP), STAT3 (1:1000, CST, 4904S), P‐STAT3 (1:1000, CST, 9145S), JAK2 (1:1000, CST, 3230S), P‐JAK2 (1:1000, CST, 3771S) and GAPDH (1:1000, CST, USA, 2118). After washed by TBST, the membranes were incubated with HRP‐linked secondary antibody (1:2000, CST, USA, 7074) for 1 hour at room temperature. The proteins in membranes were visualized using chemiluminescence kit (Thermo Fisher Scientific).

### 2.10 Statistical analysis

All values were analyzed by GraphPad Prism and recorded as the means ± SD. Student's two‐tailed *t* test was used to determine the statistical significance. *P* values <.05 were considered statistically significant. *P < .05; **P < .01; ***P < .001.

## 3 RESULTS

### 3.1 Oxysophocarpine inhibited the proliferation and increased the apoptosis of Hepa1‐6 and HepG2 cells

**FIGURE 1:**
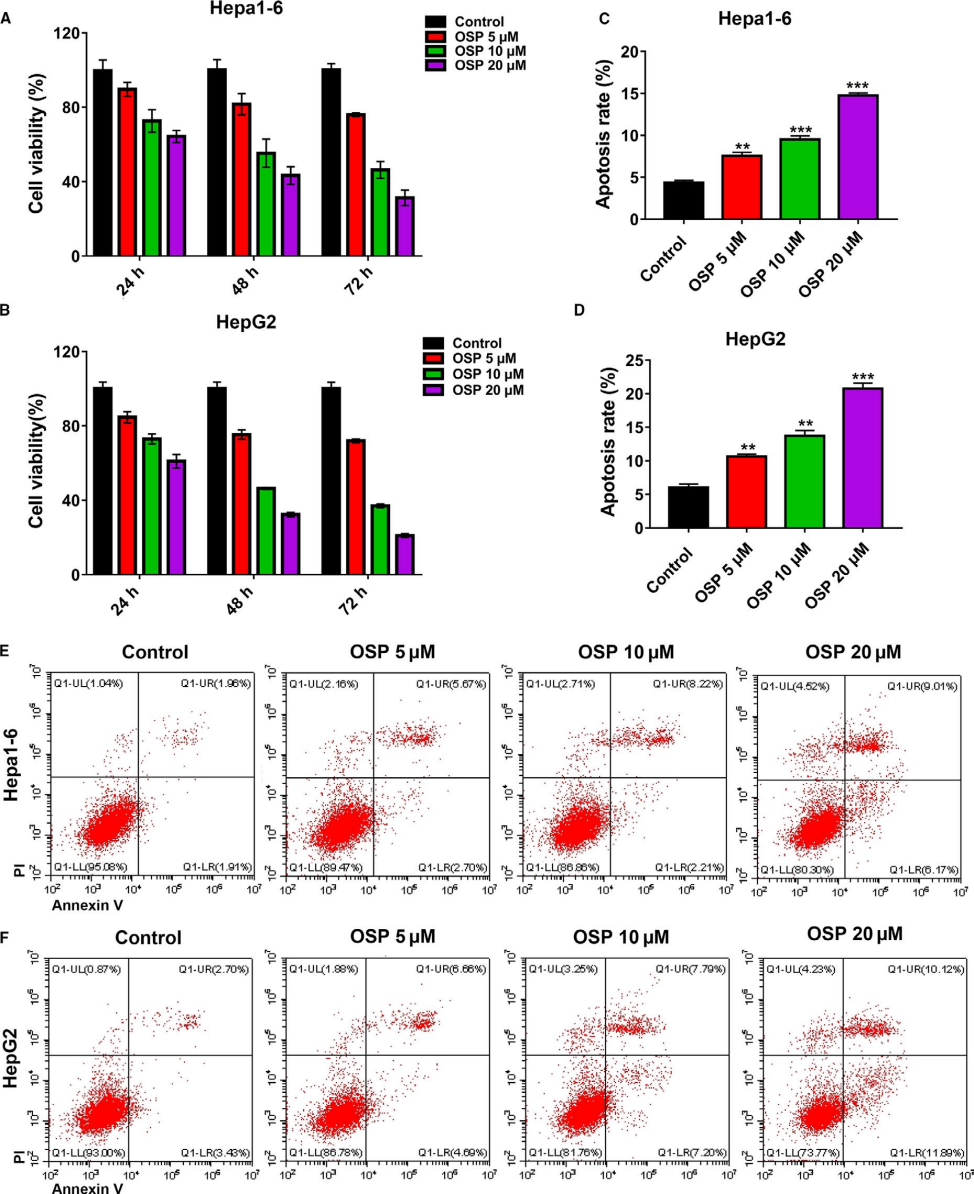
Oxysophocarpine inhibited the proliferation and increased the apoptosis of Hepa1‐6 and HepG2 cells. Hepa1‐6 and HepG2 cells were treated with different dose of Oxysophocarpine (0, 5, 10, and 20 µmol/L) for 24, 48, or 72 h and the inhibition rate was determined by CCK‐8 assay. A and B, Growth inhibition effects of Oxysophocarpine (OSP) on Hepa1‐6 and HepG2 cells. C and D, Effect of Oxysophocarpine on the apoptosis of Hepa1‐6 and HepG2 cells, Hepa1‐6, and HepG2 cells were incubated with various concentration (0, 5, 10, and 20 µmol/L) of OSP for 24 h and the apoptotic effect of Oxysophocarpine on Hepa1‐6 and HepG2 cells was determined by the Annexin‐V/PI staining assay through flow cytometry. E and F, The representative gating images of Oxysophocarpine on the Hepa1‐6 or HepG2 cells apoptosis were shown. Data are presented as means ± SD. *P < .05; **P < .01; ***P < .001

To investigate the effect of Oxysophocarpine treatment on the growth of HCC cells, HepG2, and Hepa1‐6 cells were treated with Oxysophocarpine at different concentration (0, 5, 10, and 20 µmol/L) for 24, 48, and 72 hours. The results of CCK‐8 assays indicated that Oxysophocarpine significantly inhibited the proliferation of Hepa1‐6 (Figure 1A) and HepG2 cells (Figure 1B) with a time and dose‐dependent manner. To further study the effects of Oxysophocarpine on Hepa1‐6 and HepG2 cells apoptosis, Annexin‐V‐FITC/PI staining assay was performed. The flow cytometry assays shown that the apoptosis rate of Hepa1‐6 (Figure 1C,E) and HepG2 cells (Figure 1D,F) were obviously increased in a dose‐dependent manner after Oxysophocarpine treatment for 24 hours. These results indicated that Oxysophocarpine could distinctly inhibit the proliferation and increase the apoptosis of HepG2 and Hepa1‐6 cells in vitro.

### 3.2 Oxysophocarpine suppressed the migration of Hepa1‐6 and HepG2 cells

**FIGURE 2:**
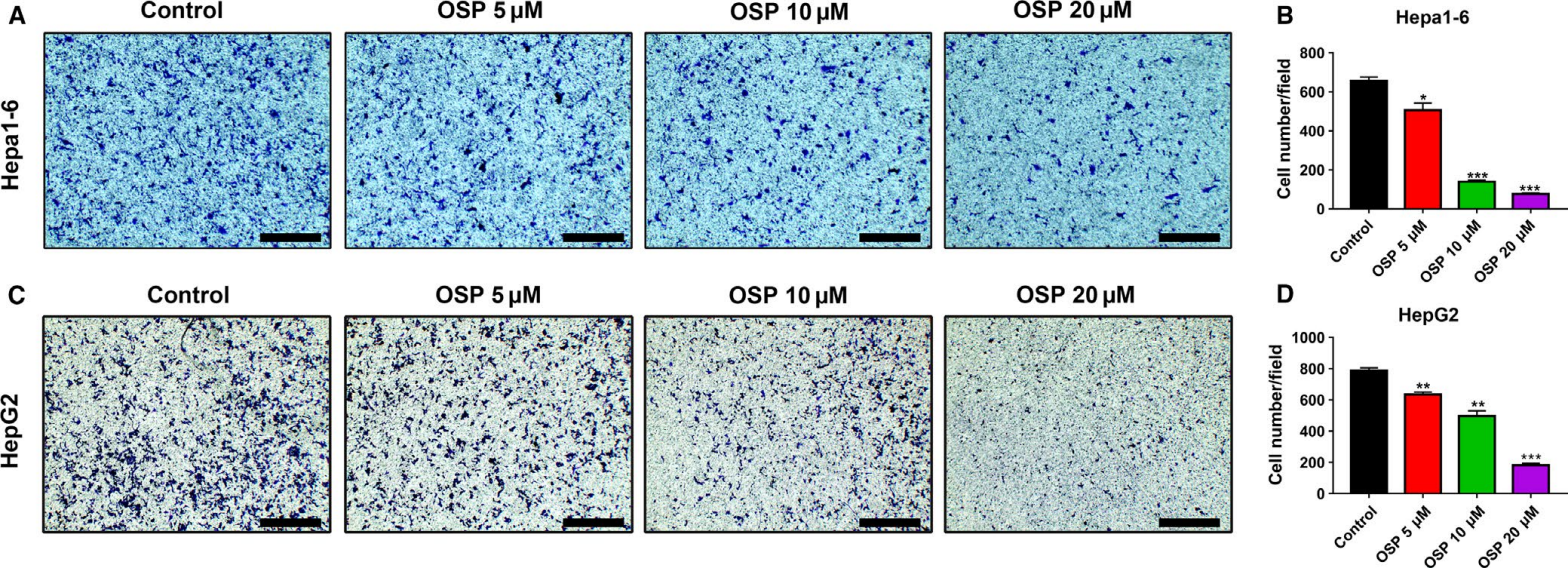
Oxysophocarpine suppressed the migration of Hepa1‐6 and HepG2 cells. A and C, Representative images of the migration ability of Hepa1‐6 and HepG2 cells were shown after previously treated with different dose Oxysophocarpine (0, 5, 10, and 20 µmol/L) for 24 h. B and D, The numbers of migrated Hepa1‐6 and HepG2 cells were quantified. Data are presented as means ± SD. *P < .05; **P < .01; ***P < .001

We further investigated the effect of Oxysophocarpine treatment on the migration of liver cancer cell lines using Trans‐well assays. HepG2 and Hepa1‐6 cells were previously treated with different doses of Oxysophocarpine (0, 5, 10, and 20 µmol/L) for 24 hours and then seeded into trans‐well plates. The results showed that the migration capacity of Hepa1‐6 cells (Figure 2A,B) and HepG2 cells (Figure 2C,D) was significantly inhibited after Oxysophocarpine treatment in a dose‐dependent manner compared with control group. This finding suggested that Oxysophocarpine suppressed the migration of Hepa1‐6 and HepG2 cells.

### 3.3 Oxysophocarpine sensitized anti‐Lag‐3 immunotherapy effect against HCC in vitro and in vivo

**FIGURE 3:**
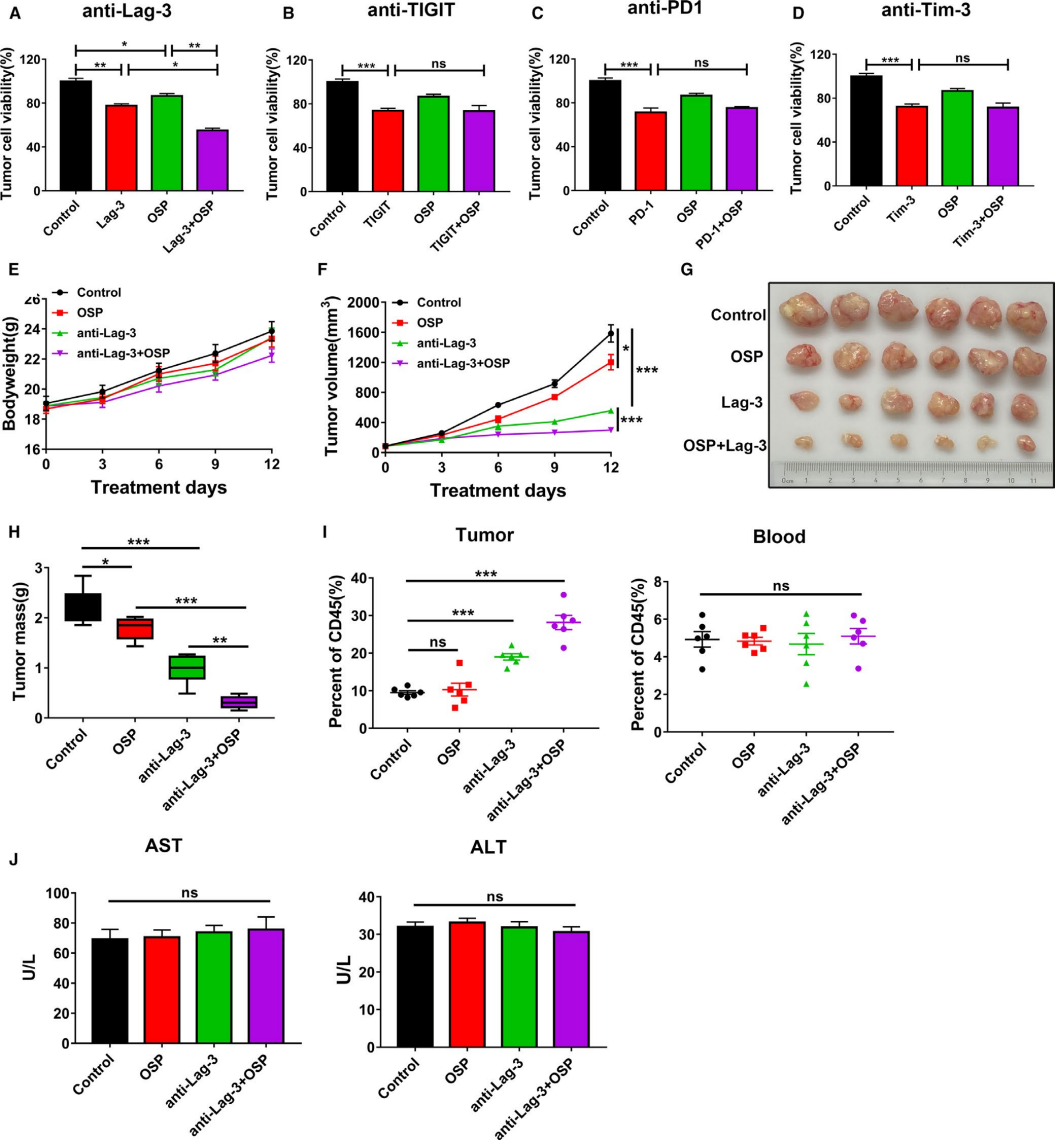
Oxysophocarpine sensitized anti‐Lag‐3 immunotherapy effect against HCC in vitro and in vivo. A‐D, Oxysophocarpine sensitized the Lag‐3 immunotherapy effect of CD8^+^ T against Hepa1‐6 cells. Hepa1‐6‐luciferase labeled cells were treated with 5 µmol/L Oxysophocarpine for 24 h first and then cocultured with CD8^+^ T cells with 10 μg/mL anti‐Lag‐3 (A), anti‐TIGIT (B), anti‐PD1 (C), and anti‐Tim3 antibody (D) for another 48 h. The viability degree of Hepa1‐6 cells was determined by fluorescence values through fluorescence microplate reader. E‐J, C57BL/6 mice were burdened with Hepa1‐6 subcutaneous tumors first and divided into different treatment group (n = 6). Mice were treated with 50 mg/kg Oxysophocarpine (daily, intraperitoneally), anti‐Lag‐3 antibody (100 μg/per mouse, every 5 d, intraperitoneally) or Oxysophocarpine combined with anti‐Lag‐3 antibody. The mouse bodyweight changes (E), tumor growth change curves (F), tumor images (G), and tumor mass (H) were measured during the treatment period. I, The ratio of CD8^+^ T cells in tumor tissues or blood was detected by flow cytometry. J, The serum levels of AST and ALT were quantified after indicated treatments. Data are presented as means ± SD. *P < .05; **P < .01; ***P < .001

Immunotherapy has becoming a promising treatment for HCC.^5^ To assess whether the Oxysophocarpine‐educated tumor cells were more sensitized to the cytotoxicity function of CD8^+^ T cells with immune checkpoint blockade, luciferase‐labeled Hepa1‐6 cells and CD8^+^ T cells coculture assays were performed. To exclude the distinct killing effect of Oxysophocarpine on Hepa1‐6 cells, we chose low dose Oxysophocarpine (5 µmol/L) as treatment dose (Figure 1), CD8^+^ T cells co‐cultured with Oxysophocarpine‐treated Hepa1‐6 cells with the ratio of 5:1 and 10 μg/mL Lag‐3, PD‐1, TIGIT, or Tim‐3 antibody was added into coculture system. After 48 hours, Oxysophocarpine‐educated Hepa1‐6 cells were more vulnerable to CD8^+^ T cells cytotoxicity function with Lag‐3 blockade of CD8^+^ T cells (Figure 3A), whereas Oxysophocarpine treatment had a little effect of CD8^+^ T cells cytotoxicity function against Hepa1‐6 cells with anti‐TIGIT (Figure 3B), anti‐PD‐1 (Figure 3C), or anti‐Tim‐3 (Figure 3D) blockade. The results revealed that Oxysophocarpine may sensitize the anti‐Lag‐3 immunotherapy effect of CD8^+^ T against HCC in vitro.

To further confirm the effect of Oxysophocarpine treatment alone and combined with anti‐Lag‐3 immunotherapy against HCC in vivo, C57BL/6 mice burdened with Hepa1‐6 subcutaneous tumor were used. Oxysophocarpine treatment or combination treatment shown little effect on the bodyweight changes or liver function including aspartate aminotransferase (AST) and alanine aminotransferase (ALT) of mice (Figure 3E,J), which indicated the safety of Oxysophocarpine treatment. Here, Oxysophocarpine or anti‐Lag‐3 treatment alone inhibited the tumor growth (Figure 3F,G) and tumor weight (Figure 3H), however, the combination treatment exerted the most effective therapeutic effect against HCC (Figure 3F‐H). Furthermore, the combination treatment could obviously increase the ratio of CD8^+^ T cells in tumor microenvironment, whereas had little influence on CD8^+^ T cells in mouse blood (Figure 3I). Based on the in vitro and in vivo results, we hypothesized that Oxysophocarpine could suppress the hepatocellular carcinoma growth and sensitize the therapeutic blockade of anti‐Lag‐3.

### 3.4 Oxysophocarpine decreased the FGL1 expression through downregulating IL‐6‐mediated JAK2/STAT3 signal pathway

**FIGURE 4:**
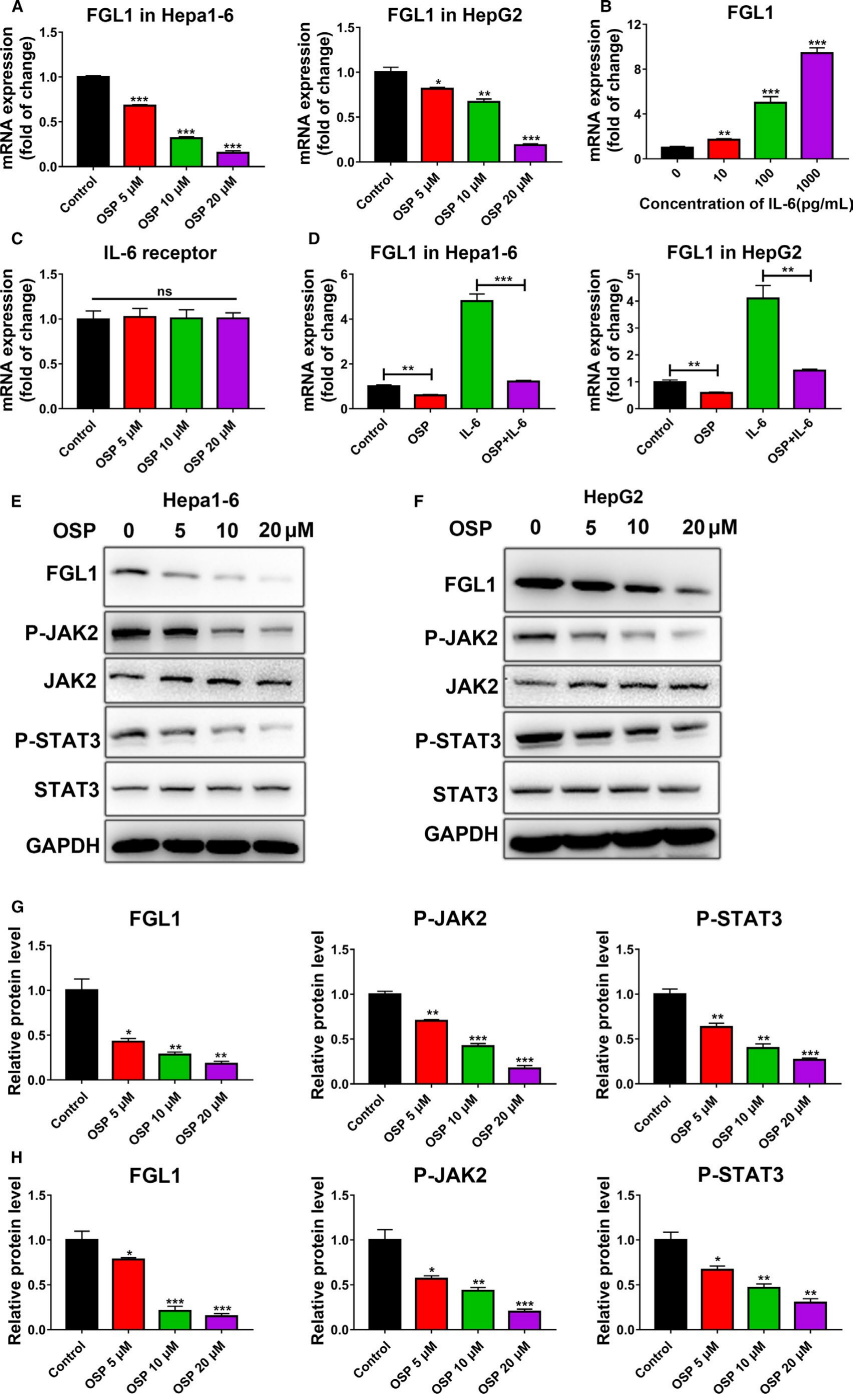
Effect of Oxysophocarpine on the expression of FGL1 and JAK2/STAT3 signal pathway in HepG2 or Hepa1‐6 cells. A, The mRNA expression of FGL1 in Hepa1‐6 and HepG2 cells after different dose Oxysophocarpine treatment (0, 5, 10, and 20 µmol/L) for 24 h. B, The mRNA expression of FGL1 in HepG2 cells treated with IL‐6 at the concentration of 0, 10, 100, or 1000 pg/mL for 24 h. C, The mRNA expression of IL‐6 receptor in HepG2 cells after Oxysophocarpine treatment for 24 h. D, Hepa1‐6 or HepG2 cells were treated with 100 pg/ml IL‐6 and 10 µmol/L Oxysophocarpine treatment for 24 h. The mRNA expression of FGL1 in Hepa1‐6 or HepG2 cells were detected. E and F, The amount of FGL1, P‐JAK2, JAK2, P‐STAT3, and STAT3 proteins in Hepa1‐6 and HepG2 cells after intervened with different concentration Oxysophocarpine. G and H, The relative protein expression ratio of FGL1, P‐JAK2, and P‐STAT3 were quantified in Hepa1‐6 (G) and HepG2 cells (H). Data are presented as means ± SD. *P < .05; **P < .01; ***P < .001

Since Oxysophocarpine sensitized the Lag‐3 immunotherapy effect of CD8^+^ T against HCC and Fibrinogen‐like‐protein 1 (FGL1) was recently identified as a major ligand for the inhibitory receptor Lag‐3 that mediated CD8^+^T cells suppression.^18^ We first explored whether Oxysophocarpine treatment could affect the expression of FGL1 in liver cancer cells. Hepa1‐6 and HepG2 cells were previously treated with different dosage Oxysophocarpine for 24 hours and then the mRNA and protein expression of FGL1 in Hepa1‐6 and HepG2 cells was determined by qPCR and western blotting assays (Figure 4). The results show that Oxysophocarpine treatment decreased the FGL1 expression of Hepa1‐6 (Figure 4A,E,G) and HepG2 cells (Figure 4A,F,H) in a dose‐dependent manner. Previous studies showed that FGL1 was induced by IL‐6 in HepG2 cells in a dose dependent manner.^19^, ^20^ It is recognized that IL‐6 binds to its receptor and subsequently activate the JAK2/STAT3 signaling axis in cells.^21^, ^22^ Hence, we speculated whether Oxysophocarpine treatment downregulate IL‐6‐mediated JAK2/STAT3 activation to decrease FGL1 expression. First, we confirmed the effect of IL‐6 on inducing FGL1 expression in HepG2 cells. The mRNA expression of FGL1 were obviously upregulated in a dose dependent manner after IL‐6 treatment for 24 hours (Figure 4B). Then we further excluded the influence of Oxysophocarpine on IL‐6 receptor expression in HepG2 cells. The results showed Oxysophocarpine had no effect on the expression of IL‐6 receptor (Figure 4C). Next, we explored the effect of Oxysophocarpine on FGL1 expression in IL‐6‐stimulated Hepa1‐6 and HepG2 cells. The expression of FGL1 was significantly suppressed by Oxysophocarpine treatment, indicating Oxysophocarpine could inhibit IL‐6 induced FGL1 expression (Figure 4D). Last, we detected the protein expression of P‐JAK2, JAK2, P‐STAT3, and STAT3 in Oxysophocarpine‐treated Hepa1‐6 and HepG2 cells by western blotting assay. As shown, Oxysophocarpine decreased the expression levels of P‐JAK2 and P‐STAT3 significantly in Hepa1‐6 cells (Figure 4A,E,G) and HepG2 cells (Figure 4A,F,H).

**FIGURE 5:**
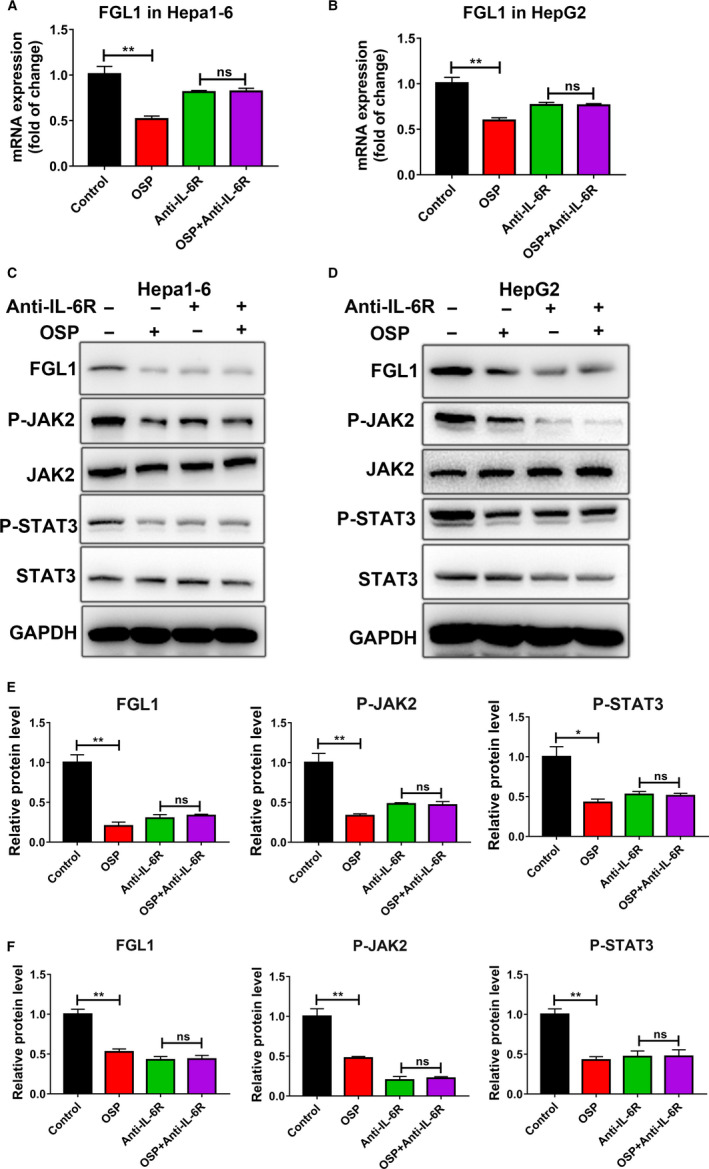
Oxysophocarpine decreased the FGL1 expression through downregulating IL‐6‐mediated JAK2/STAT3 signal pathway. A and B, Hepa1‐6 or HepG2 cells were previously treated with 5 μg/mL anti‐IL‐6R antibody for 24 h before 10 µmol/L Oxysophocarpine treatment for another 24 h. The mRNA expression of FGL1 in Hepa1‐6 and HepG2 cells were detected. C and D, The expression of FGL1, P‐JAK2, JAK2, P‐STAT3, and STAT3 proteins in Hepa1‐6 and HepG2 cells were detected after indicated treatment. E and F, The relative protein expression ratio of FGL1, P‐JAK2, and P‐STAT3 were quantified in Hepa1‐6 (E) and HepG2 cells (F). Data are presented as means ± SD. *P < .05; **P < .01; ***P < .001

To verify whether IL‐6‐mediated JAK2/STAT3 signal pathway is responsible for FGL1 suppression in Oxysophocarpine‐treated HepG2 and Hepa1‐6 cells, anti‐IL‐6 receptor antibody was used to block IL‐6 receptor in HepG2 and Hepa1‐6 cells. Hepa1‐6 or HepG2 cells were previously treated with 5μg/mL anti‐IL6R antibody for 24 hours before 10 µmol/L Oxysophocarpine treatment for another 24 hours. The results show that Oxysophocarpine combined with anti‐IL‐6R show a similar suppression level on FGL1, P‐JAK2, and P‐STAT3 expression compared to anti‐IL‐6R treatment alone in Hepa1‐6 (Figure 5A,C,E) and HepG2 cells (Figure 5B,D,F). After IL‐6 receptor was blocked, Oxysophocarpine did not show a more efficient suppression effect on FGL1, P‐JAK2, and P‐STAT3 expression which indicated Oxysophocarpine suppress IL‐6‐mediated JAK2/STAT3 signal activation to downregulate FGL1 expression. These data reflected that Oxysophocarpine decreased FGL1 expression through downregulating IL‐6‐mediated JAK2/STAT3 signal pathway in HCC cells and subsequently sensitize anti‐Lag3 therapeutic effect.

## 4 DISCUSSION

Oxysophocarpine is a bioactive alkaloid extracted from natural products, which exert various pharmacological activities.^23^ For the anti‐tumor activity of Oxysophocarpine, it has been identified that Oxysophocarpine suppresses oral squamous cell carcinoma by targeting the Nrf2/HO‐1 axis and inhibits H22 liver cancer cells growth.^11^ In this study, we found Oxysophocarpine slowed the growth of HCC and sensitized the immunotherapy of anti‐Lag‐3 in vivo or in vitro. Key findings were as follows. First, Oxysophocarpine could distinctly suppress the growth and induce apoptosis of HCC cells (Hepa1‐6 and HepG2). Second, Oxysophocarpine inhibited the migration ability of HCC cells. Third, Oxysophocarpine sensitized the immunotherapy effect of anti‐Lag‐3 without side effect, whereas had a little effect with TIGIT, PD‐1, or Tim‐3 blockade against HCC cells. Finally, Oxysophocarpine decreased the FGL1 expression through downregulating IL‐6‐mediated JAK2/STAT3 axis to sensitize the anti‐Lag‐3 immunotherapy effect against HCC. Overall, these results indicated that Oxysophocarpine reduced HCC growth and sensitized the anti‐Lag‐3 immunotherapy effect through decreasing FGL1 expression.

HCC is an aggressive malignancy with limited effective treatments and ranks as the second most lethal tumor next to pancreatic cancer.^24^ In clinical, only two systemic drugs, sorafenib and lenvatinib, have been approved by FDA for advanced HCC as the first‐line treatment.^1^ For sorafenib, only about 30% of HCC patients benefit from treatment with limited efficacy due to the individual differences, tumor heterogeneity, immunosuppressive microenvironment, intrinsic and acquired chemotherapy resistance.^25^ As a result, developing a potential drug is necessary against HCC. Immunotherapy has been recognized as an effective approach for cancer treatment.^26^ The already identified immune checkpoints mainly include PD‐1, CTLA‐4, Lag‐3, Tim‐3, and TIGIT.^8^ However the clinical efficiency is varied due to individual's response, different immune‐targets and tumor types.^5^ Hence, more exploration is needed in immune‐based therapies.

Lag‐3 is a transmembrane protein primary found on activated T cells and represent a function exhaustion of CD8^+^ T cells similar to PD‐1 in response to cancers.^18^ FGL1 is a protein secreted by hepatocytes as a mitogen for liver cell proliferation.^27^ However, FGL1 is normally released by the liver in low levels but by cancer in high levels.^18^, ^27^ FGL1 was found to be over‐expressed in human liver cancer as a major ligand of Lag‐3 which is responsible for its T‐cell inhibitory function, and had a link with a poor prognosis and therapeutic outcome.^18^, ^19^, ^20^ Previous researches find that the expression of FGL1 is obviously upregulated in HepG2 cell cocultured with IL‐6 and the plasma levels of FGL1 is enhanced in mice after intraperitoneal injection of recombinant IL‐6.^19^, ^20^ IL‐6 is a pleiotropic cytokine linked to pro‐inflammation, regeneration, and tumor development.^28^ IL‐6 promotes the proliferation, survival, and metastasis of cancer cells to enhance angiogenesis.^29^ Moreover IL‐6 promotes the polarization of macrophages to a M2 phenotype in the inflamed liver to play pro‐tumor effect.^30^, ^31^ However, IL‐6 also plays key role in the activation, proliferation, and survival of CD8^+^ T cells to paly anti‐tumor function.^22^ IL‐6 is recognized to binds its receptor and subsequently activate JAK2/STAT3 signaling axis, which play a role as a risk factor in HCC.^31^, ^32^ Here, we identified that Oxysophocarpine sensitized the Lag‐3 immunotherapy effect of CD8^+^ T against HCC in vivo and in vitro. Oxysophocarpine decreased the FGL1 expression through downregulating IL‐6‐mediated JAK2/STAT3 axis.

In summary, this study systematically revelated the anti‐growth and anti‐migration properties of Oxysophocarpine against HCC. Oxysophocarpine sensitized the anti‐Lag‐3 immunotherapy effect of CD8^+^ T cells against HCC by decreasing FGL1 expression. Mechanistic studies releaved the effect of Oxysophocarpine on FGL1 was depended on IL‐6‐mediated JAK2/STAT3 pathway. These findings provide preclinical evidences to show Oxysophocarpine may have important applications in HCC treatment and can be used as a combination treatment with anti‐Lag‐3 immunotherapy.

## CONFLICT OF INTEREST

The authors have no conflicts of interest.

## AUTHOR CONTRIBUTIONS

Jianchu Wang, Wang Wei, Qianli Tang, and Jian Pu conceived and designed the experiments; Jian Pu supervised this project; Jianchu Wang, Wang Wei, and Qianli Tang performed the experiments and wrote the original draft; Libai Lu, Zongjiang Luo Wenchuan Li, and Yuan Lu participated in the data collation.

## ETHICAL APPROVAL

This study was approved by Affiliated Hospital of Youjiang Medical University for Nationalities.

## Data Availability

The data that support the findings of this study are available from the corresponding author upon reasonable request.
